# Supplementary material for: Preliminary Assessment of Burn Depth by Paper-Based ELISA for the Detection of Angiogenin in Burn Blister Fluid—A Proof of Concept
Source: Diagnostics (Basel). 2020 Feb 27;10(3):127. doi: 10.3390/diagnostics10030127 (PMC7151184; doi:10.3390/diagnostics10030127)
Supplement: Supplementary file 1 [file diagnostics-10-00127-s001.zip › Supporting Information-20200212.docx]

**Rapid Assessment of Burn Depth by Paper-based ELISA for the Detection of Angiogenin in Burn Blister Fluid**

**Shin-Chen Pan^1,2^ M.D., Ph.D., Yao-Hung Tsai^3^ B.S., Chin-Chuan Chuang^3^, Chao-Min Cheng^3,*^ Ph.D.**

^1^Department of Surgery, Section of Plastic and Reconstructive Surgery, National Cheng Kung University Hospital, College of Medicine, National Cheng Kung University, Tainan, Taiwan

^2^International Center for Wound Repair and Regeneration, National Cheng Kung University, Tainan, Taiwan

^3^Institute of Biomedical Engineering and Frontier Research Center on Fundamental and Applied Sciences of Matters, National Tsing Hua University, Hsinchu, Taiwan

**Figure S1.** Anti-angiogenin antibody concentration modification. The results using primary antibody at 3 different concentrations; 3 µg/mL, 2 µg/mL, and 1 µg/mL. The data indicate that 1 µg/mL of anti-angiogenin antibody produced the best effect. Samples 1 to 3 were collected from DPTB blister fluids. Healthy human blood serum was the control.

**Figure S2.** Secondary antibody concentration modification. The result of secondary antibody at 3 different concentrations,; 0.05 µg/mL, 0.04 µg/mL, and 0.033 µg/mL. The data indicate that 0.033 µg/mL of secondary antibody produced the best effect. Samples 1 to 3 were collected from DPTB blister fluids. Healthy human blood serum was the control.

Figure S3. Blocking step modification. The result of BSA blocking solutions at 3 different concentrations; 1%, 0.5%, and 0.1%. The data indicate that 1% BSA produced the best effect. Samples 1 to 3 were collected from DPTB blister fluids. Healthy human blood serum was the control.

**Figure S4.** Washing volume modification. The results from using 3 different washing volumes; 15 µL, 10 µL, and 5 µL. The data indicate that 15 µL produced the best effect. Samples 1 to 3 were collected from DPTB blister fluids. Healthy human blood serum was the control.

**Figure S5.** Washing times modification. The results from two different washing times,; 3 times (5 µL + 5 µL +5 µL) and 2 times (5 µL + 10 µL). The data indicate that the method with the best effect was to wash 2 times. Samples 1 to 3 were collected from DPTB blister fluids. Healthy human blood serum was the control.

**Figure S6.** Calibration curve of angiogenin of plate ELISA.

**Figure S7.** Primary antibody functional test. The P-ELISA is tested either with or without the treatment of primary antibody. The data shows a significant color development when treatment with primary antibody. (mean ± S.D, *P < 0.05)

**Table S1.** Patient demographic data

| Burn depth | Gender | Age | TBSA% | Burn area | Aspiration time  (hr) | Angiogenin ( man±SD ,  ng/mL) | VEGF  (man±SD, ng/mL) | pH |
| --- | --- | --- | --- | --- | --- | --- | --- | --- |
| SPTB | M | 59 | 1 | Left Hand | 20 | 57.3±17.6 | 29.5±15.1 | 9 |
|  | F | 81 | 5 | Right Hand | 19 | 176.5±17.7 | — | 8 |
|  | M | 27 | 10 | Right Hand | 39 | 85.7±16.1 | 41.2±13.2 | 9 |
|  | M | 45 | 6 | Left Hand | 16 | 157.3±23.6 | 23.9±23.7 | 8 |
|  | F | 48 | 1 | Left Foot | 23 | — | 31.3±10.5 | 8 |
|  | F | 58 | 1 | Left Hand | 24 | — | — | — |
| DPTB | F | 41 | 5 | Left Foot | 22 | 202.3±65.3 | 34.7±3.3 | 9 |
|  | M | 39 | 30 | Buttock | 14 | 587.3±68.1 | 46.8±10.1 | 9 |
|  | F | 8 | 10 | Left Thigh | 19 | 160.7±14.4 | 27.1±5.1 | 9 |
|  | M | 52 | 13 | Left Hand | 65 | 142.3±37.9 | 89.0±43.9 | 9 |
|  | F | 48 | 1 | Right Foot | 23 | 554±73.7 | — | 8 |
|  | M | 50 | 10 | Right Hand | 22 | 342.3±45.4 | 38.2±4.6 | 8 |

**SPTB: superficial partial thickness burn**

**DPTB: deep partial thickness burn, TBSA: total body surface area**
